# Surgical Management of Vertical Root Fracture (VRF): A Report of Two Cases

**DOI:** 10.7759/cureus.112045

**Published:** 2026-07-04

**Authors:** Maryam Kuzekanani

**Affiliations:** 1 Department of Endodontics, Endodontology Research Center, Kerman University of Medical Sciences and Health Services, Kerman, IRN

**Keywords:** case report, management, surgical, treatment, vertical root fracture

## Abstract

Vertical root fracture (VRF) is one of the most challenging complications in endodontic treatment. Diagnosing VRF, especially in the early stages, can be difficult, and it is often recognized only during surgical procedures or on high-quality cone beam computed tomography (CBCT) scans. Cracked teeth and VRF represent a rising concern in endodontic management. Tooth extraction for single-rooted teeth and root amputation for multi-rooted teeth are still the last solutions suggested. The most important issue with VRFs is that the fracture line lies below the level of the epithelial attachment, allowing salivary microorganisms to penetrate the fracture space, thereby making the likelihood of fracture repair and the resulting lesion very poor. This paper aims to review the etiology, diagnosis, clinical symptoms, and present the surgical management of two cases of VRF.

## Introduction

A vertical root fracture (VRF) is a fracture along the longitudinal axis of the tooth that extends from the root canal to the periodontium. Thus, a vertical fracture involves the cementum, dentine, and pulp of the tooth. The site of fracture initiation may be the crown, apex, or a point between the crown and apex [1,2]. VRFs are classified into two categories based on their extent and boundaries: complete fractures, which involve both opposing surfaces of the root, and incomplete fractures that involve only one surface of the root. VRFs usually, but not always, occur completely and extend from one surface of the root towards the canal and the opposite surface [3]. These fractures are often observed in mandibular molars on the mesial or distal root surface in a mesiodistal or opposite direction. The direction of the fracture can also be from the buccal to the lingual surface or from the lingual to the buccal, but overall, buccolingual fractures are more prevalent [4]. Symptoms resulting from VRFs indicate the fracture's extent and location. It should be noted that these fractures may involve the entire length of the root, or include only a portion of the root length in the coronal or apical direction, and finally extend along the crown as well as the root [5].

Etiology

Endodontic procedures may be one of the causes of VRFs. Excessive widening of the root canal, habits such as not following the correct anatomical form of the root canal, and using inappropriate instruments can lead to weakening of the canal walls. Therefore, before starting instrumentation, we should pay attention to points such as the size and length of the root as well as the anatomical form of the root. Using excessive force to compact the gutta-percha is another important factor that can lead to VRFs. The most common causes of the VRFs are trauma, restorative procedures, and endodontic iatrogenic errors.

Trauma

VRFs may result from sudden trauma or from gradual, chronic trauma associated with occlusal disharmonies. Teeth with incompletely formed roots usually do not develop root fractures. Root fractures occur more frequently in teeth with fully formed or nearly complete roots. Trauma to young teeth with incompletely formed roots usually results in avulsion (displacement of the tooth from the socket), partial loosening or subluxation, and may eventually lead to complete loosening or luxation [3].

Restorative procedures

Restorative procedures that may cause VRFs include excessive force when placing a pin or post and excessive force when placing an intracoronal inlay. Many researchers believe that root canal-treated teeth are more prone to fracture for one or both of the following reasons: dehydration or drying of the dentin, and removal of dentin during preparation of the access cavity to the pulp chamber and the use of instruments inside the canal. To prevent this fracture, an intradentally placed support structure, such as a pin or post, is often used and is usually connected to a core to enhance post stability. Posts are divided into three main categories based on the shape and method of creating the retention: tapered, parallel, and threaded posts. Posts are also used in various groups of pulpless teeth: a tooth with a healthy and intact crown, a tooth that has lost part of its crown, and a tooth that has lost its entire crown. When placing a post, one should not use too much force. Experience has shown that screwed posts lead to VRFs more than screwless posts. Although many researchers have mentioned the advantages of using posts and cores, the disadvantages of their use have received less attention [6-8].

Major disadvantages of posts and cores

Using posts that are thicker than the root diameter may weaken the root and ultimately cause its fracture or perforation. If the clinical crown length is too short, the use of a post and core may increase lateral forces on the tooth root. An increase in these forces can cause root fracture, bone loss, or tooth loss. Sometimes, the collar areas of cores extend below the gum, leading to chronic inflammatory reactions that result in alveolar bone loss, severe tooth loss, and, unfortunately, tooth loss [9].

Endodontic iatrogenic errors

Endodontic procedures can cause VRFs. Excessive widening of the root canal, failure to follow the correct anatomical form of the canal, and use of inappropriate instruments can weaken the canal walls. Therefore, before starting instrumentation, attention should be paid to factors such as the size and length of the root, as well as its anatomical form. Using excessive force to compact the gutta-percha is another important factor that can lead to VRFs [8].

Diagnosis

Diagnosing VRFs and cracked teeth begins with the chief complaint and dental history, as well as a visual clinical examination. The patient’s chief complaint may involve pain when chewing, biting down, or upon release, or hearing a cracking sound while chewing hard foods such as a cherry stone [10]. Diagnosis of a VRF is challenging, particularly in the early stages. It may become easier after several weeks or months, once the tissues surrounding the root have changed or become damaged. If the root fragments have separated, diagnosis is straightforward; however, in such cases, the prognosis for treatment is very poor. Even in many cases where fractures are easily visible, there may be no clear radiographic appearance during surgery or tooth extraction. This suggests that sometimes the surface exposed to radiation is not sufficiently close to the fracture line, making radiographic diagnosis of VRF feasible [11]. Rud and Omnell, in their study of a VRF specimen after tooth extraction and lateral alignment of the fracture fragments, found that the fracture was clearly visible on radiographs only when the X-ray beam was directed at the tooth at an angle of four degrees to the fracture line. In such cases, a vertical radiolucent line or area parallel to the fracture line may be observed [12]. It is important to note that even when the radiation is applied in a superimposed position, it is still possible that the fracture may not be visible on the radiograph due to the presence of post pins and other radiopaque root canal filling materials [7]. 

Clinical signs

Most signs and symptoms of VRFs may also occur with conditions such as pulp necrosis or endodontic treatment failures. These signs include brief episodes of cold sensitivity, peri-radicular lesions, sinus tract formation (usually not present in the early stages), sensitivity to trauma, swelling, tooth mobility, pain, and deep periodontal pockets. Observing these signs and symptoms can help identify the presence of a VRF, but they are not sufficient to definitively diagnose the fracture or its specific cause. Proposed techniques for diagnosing VRFs include directing a fiber-optic light horizontally on the gingival sulcus and perpendicular to the tooth in a dark room, from various angles and directions. In the presence of a VRF, a continuous dark line, often in the mesiodistal direction in posterior teeth, is visible against the bright background of the pulp floor. The diagnosis is more reliable if previous crown fillings are removed before the test. Current recommendations advise using a surgical operating microscope to inspect the tooth, as the naked eye often cannot distinguish the details of VRFs or cracks. When transilluminating the tooth surface from different directions, a minute crack may sometimes be seen under magnification, and staining the suspicious site with a dye helps to identify the crack line [7]. The recommended clinical tests for diagnosing a cracked or fractured tooth are as follows: soft tissue examination to detect sinus tracts, percussion, palpation, biting or chewing tests, vitality tests (thermal tests and electric pulp testing), restoration removal for improved visualization, periodontal probing using flexible plastic probes, and restoration removal if necessary for complete visualization [11].

## Case presentation

Case 1

Patient 1 was a 24-year-old female without any remarkable systemic disease referred to the Endodontist with a chief complaint of spontaneous pain in the right mandibular first molar for the past month. Clinical examination revealed tenderness to percussion and a grade 1 mobility on periodontal testing. The patient had no significant medical history. The pre-operative periapical radiograph showed inadequate root canal treatment, periodontal ligament involvement, and both lateral and apical radiolucencies. The previous endodontic treatment had failed due to a lack of apical seal, strip perforation, and separation of an instrument within the mesiobuccal root (Figure 1A). Therefore, endodontic surgery was performed on the affected tooth. A vertical flap was designed and raised over the mesiobuccal root surface. After exposing the mesiobuccal root, the root tip was amputated with a bevel towards the buccal surface (Figure 1B). A complete VRF extending from the buccal to the lingual surface of the mesial root was observed on the bevelled surface of the amputated mesial root, involving both the mesiobuccal and mesiolingual canals (Figure 2). As the prognosis for the mesial half of the tooth was poor, a hemisection was ultimately performed (Figure 1C).

**Figure 1:**
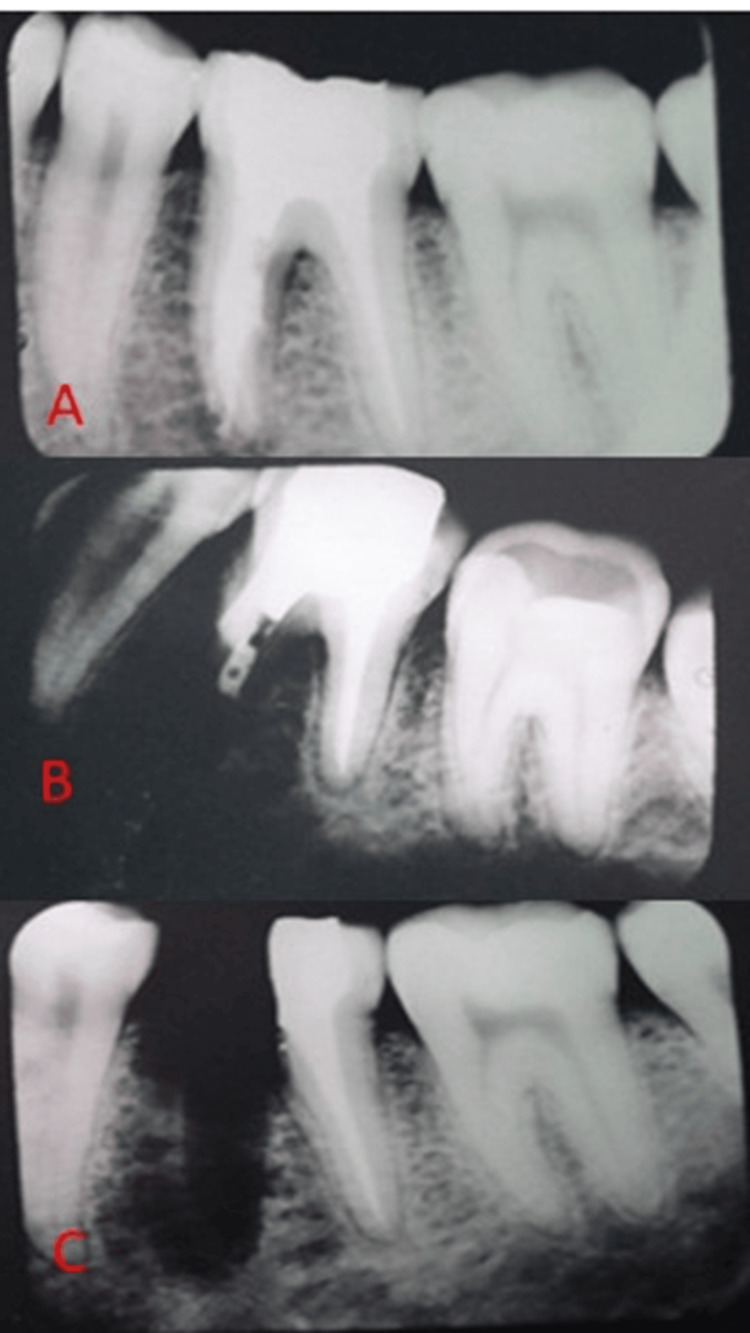
A: First radiograph of the patient showing a poor root canal treatment on the first mandibular molar, with lack of seal, strip perforation and separated instrument in mesiobuccal canal. B: Root amputation with a bevel toward buccal aspect. C: Hemisection of the molar tooth.

**Figure 2:**
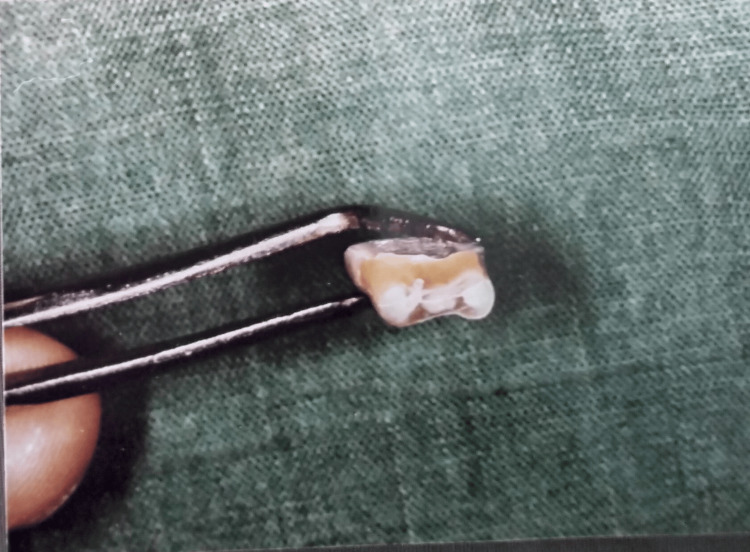
Photograph of the vertical fracture in the mesial root of the mandibular first molar (case 1). As can be seen, the above vertical fracture is complete and involves both the buccal and lingual surfaces of the root and both the mesiobuccal and mesiolingual canals.

Case 2

Patient 2 was a 34-year-old woman without any systemic disease history who was referred to the Endodontist with discomfort in her right maxillary first molar. The pain was spontaneous and mild, and was aggravated by chewing or biting on a cotton roll. The tooth had previously undergone root canal treatment, and its roots were filled with gutta-percha and sealer. On clinical examination, there was no deep pocket around the tooth. Radiographs showed that the mesiobuccal root canal was short-filled. As the patient complained of sensitivity to hot and cold foods, it was suspected that some vital tissue remained in the tooth. Therefore, the amalgam filling of the crown and the obturation of the mesiobuccal canal were removed, and the working length of the mesiobuccal canal was corrected using a size 10 file and RC-Prep (Premier Dental Products Company, Plymouth Meeting, PA, USA). It was instrumented again to the correct length. Throughout these procedures, the patient continued to experience pain, which increased day by day until she insisted on the extraction of the tooth. Ultimately, extraction was agreed upon. On visual inspection of the extracted tooth, a crack was observed on the surface of the palatal root. To confirm the presence of a VRF, the palatal root was removed, and a methylene blue dye solution was injected into it. The solution penetrated the fracture line, confirming the diagnosis. A photograph of the cracked root is seen in Figure 3. The cause of this crack or incomplete VRF may be excessive forces of spreaders or pluggers during lateral or vertical condensation of guttapercha in the past, and unsuccessful root canal treatment.

**Figure 3:**
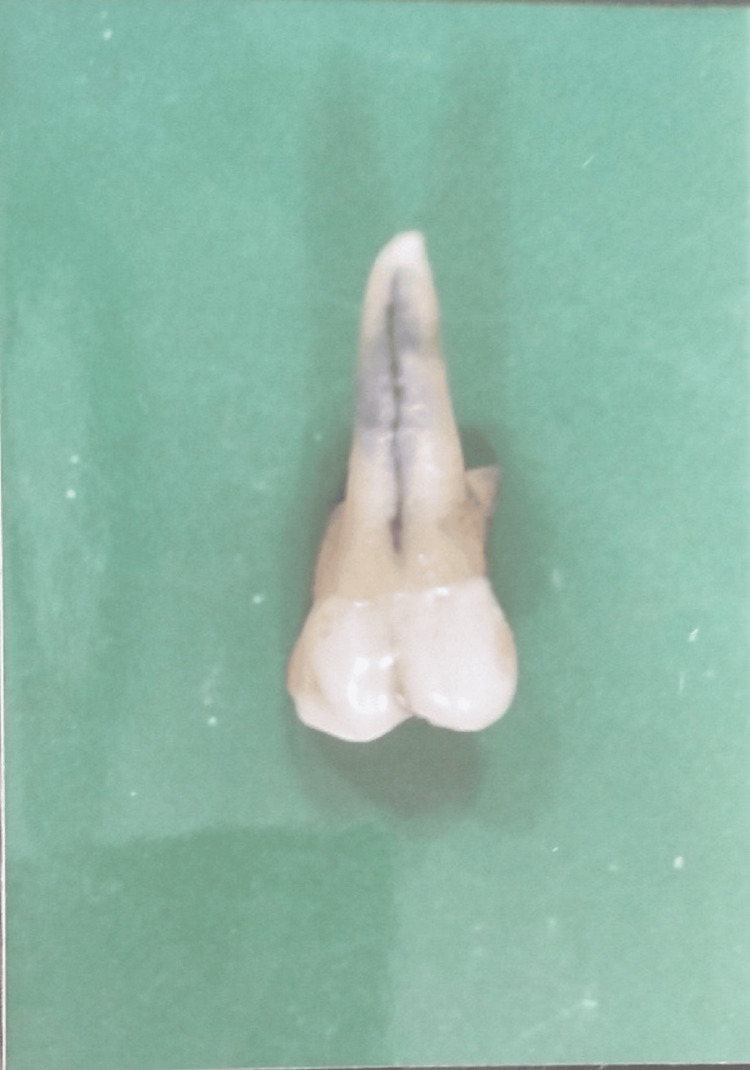
The image above from case 2 shows the palatal root of the maxillary first molar with an incomplete vertical root fracture. This fracture involves only one root surface and is stained with methylene blue.

## Discussion

Most unexpected and surprising treatment failures in teeth undergoing root canal treatment are due to VRFs. VRFs may be caused by trauma, iatrogenic errors, and pathological procedures. Iatrogenic errors include excessive removal of tooth structure during access cavity preparation to the pulp, biomechanical preparation, over-enlargement of root canals, use of rotary instruments - especially in curved areas of the root canal - excessive force used to place posts, cores, and gold castings, and excessive force applied during gutta-percha compaction in the lateral condensation technique. The minimum force required to cause a VRF is 1.5 kg for small anterior teeth and 7.2 kg for teeth with larger molar roots. It has been demonstrated that the more tapered the canal preparation, the greater the force distribution on the canal walls, resulting in a denser filling and a reduced risk of vertical fracture. Conversely, if the canal walls are prepared in parallel, the spreader force is concentrated at a single point, causing root fracture at that location [13]. Diagnosis and treatment of VRFs remain challenging in endodontic practice. Although numerous clinical and radiographic signs have been proposed for diagnosing VRFs, definitive diagnosis is often only possible through surgery and direct observation of the root surface, as these signs are also present in many other conditions, including pulp necrosis. The use of a vertical flap is preferable to other types of flaps, including modified semilunar flaps, because the vertical flap allows for viewing all parts of the root surface, and also allows for partial root resection without damaging the edges of the incision. If the flap used to diagnose vertical fractures is short, it will be difficult to diagnose fractures that originate in the apical region and do not reach the gingival sulcus. In addition, the root apex should be beveled towards the operator to facilitate diagnosis of the fracture and its extent. This beveling also enables the diagnosis of lingual fractures and cracks. As mentioned, deep periodontal pockets are one of the most important clinical signs associated with VRFs, and the presence of two deep periodontal pockets on two opposite root surfaces is a pathognomonic sign of VRFs. However, this finding has high diagnostic value only in a mouth without periodontal disease and is not a useful guide in a mouth with numerous pockets on different surfaces of multiple teeth [14].

Even systematic reviews and meta-analyses indicate that cone beam computed tomography (CBCT) scans are often insufficient to detect crack lines in some cases [15]. As fractured segments separate over time, diagnosis becomes easier, and extraction is often the only option for single-rooted teeth at this stage. Although preserving the intact section and removing the cracked portion is possible through root amputation, hemisection, and bicuspidization procedures, concerns remain regarding future stability and the complexity of restorative and prosthodontic procedures required to restore normal function. Unfortunately, we were unable to follow up with the first patient after prosthetic treatment for the crown, which limits the reporting of this case. Further root amputation treatments, along with follow-up to assess tooth stability in the alveolar socket, are recommended. Currently, many clinicians prefer to extract and replace affected multirooted teeth with implants, which offer specific advantages. However, many clinicians also prefer to preserve the intact natural root, which may be less expensive.

## Conclusions

There is still no definitive treatment for cracked or vertically fractured teeth. In multirooted teeth, it is possible to remove the fractured root and retain the intact one, but at the same time, the stability of the retained part and the complex crown restorations required in these cases remain challenging in dentistry. Therefore, prevention is the best approach by avoiding iatrogenic errors during restorative and endodontic procedures. Checking and correcting traumatic occlusions, as well as avoiding biting or chewing hard foods, are also important considerations to prevent cracks or vertical fractures in teeth. Further case presentations on root amputation or hemisection treatments for VRFs are recommended, along with further follow-up on their stability after their prosthetic treatments.
